# Novel Cysteine Desulfidase CdsB Involved in Releasing Cysteine Repression of Toxin Synthesis in *Clostridium difficile*

**DOI:** 10.3389/fcimb.2017.00531

**Published:** 2018-01-09

**Authors:** Huawei Gu, Yingyin Yang, Meng Wang, Shuyi Chen, Haiying Wang, Shan Li, Yi Ma, Jufang Wang

**Affiliations:** School of Biology and Biological Engineering, South China University of Technology, Guangzhou, China

**Keywords:** *clostrdium difficile*, *cdsB*, cysteine desulfidase, toxins synthesis, δ^54^

## Abstract

*Clostridium difficile*, a major cause of nosocomial diarrhea and pseudomembranous colitis, still poses serious health-care challenges. The expression of its two main virulence factors, TcdA and TcdB, is reportedly repressed by cysteine, but molecular mechanism remains unclear. The cysteine desulfidase CdsB affects the virulence and infection progresses of some bacteria. The *C. difficile* strain 630 genome encodes a homolog of CdsB, and in the present study, we analyzed its role in *C. difficile* 630Δ*erm* by constructing an isogenic ClosTron-based *cdsB* mutant. When *C. difficile* was cultured in TY broth supplemented with cysteine, the *cdsB* gene was rapidly induced during the exponential growth phase. The inactivation of *cdsB* not only affected the resistance of *C. difficile* to cysteine, but also altered the expression levels of intracellular cysteine-degrading enzymes and the production of hydrogen sulfide. This suggests that *C. difficile* CdsB is a major inducible cysteine-degrading enzyme. The inactivation of the *cdsB* gene in *C. difficile* also removed the cysteine-dependent repression of toxin production, but failed to remove the Na_2_S-dependent repression, which supports that the cysteine-dependent repression of toxin production is probably attributable to the accumulation of cysteine by-products. We also mapped a δ^54^ (SigL)-dependent promoter upstream from the *cdsB* gene, and *cdsB* expression was not induced in response to cysteine in the *cdsR*::*ermB* or *sigL*::*ermB* strain. Using a reporter gene fusion analysis, we identified the necessary promoter sequence for cysteine-dependent *cdsB* expression. Taken together, these results indicate that CdsB is a key inducible cysteine desulfidase in *C. difficile* which is regulated by δ^54^ and CdsR in response to cysteine and that cysteine-dependent regulation of toxin production is closely associated with cysteine degradation.

## Introduction

*Clostridium difficile* is a Gram-positive, spore-forming, obligately anaerobic gastrointestinal bacterium that causes antibiotic-associated diarrhea and pseudomembranous colitis (Burke and Lamont, 2014). In the past two decades, the epidemiology of *C. difficile* infection (CDI) has changed, including increases in the rate and severity of infection, which were related to the emergence of a hypervirulent strain and an increase in disease among outpatients in community settings (Merrigan et al., 2010). The number of discharged hospital patients with CDI in the United States more than doubled between 2001 (approximately 148,900 discharges) and 2005 (approximately 301,200 discharges), and current estimates suggest that more than 500,000 patients suffer CDI annually, with at least 14,000 deaths (Bagdasarian et al., 2015; Shields et al., 2015).

The pathogenesis of *C. difficile* mainly attributed to the action of the two large enterotoxins, TcdA and TcdB (Voth and Ballard, 2005; Rupnik et al., 2009; Awad et al., 2014). Apart from its regulation by the sigma factor TcdR (Mani and Dupuy, 2001; Carter et al., 2014) and the negative regulator TcdC (Matamouros et al., 2007), the synthesis of these two major toxins responds to various environmental signals and stresses, including the availability of certain carbon sources or amino acids (Karasawa et al., 1997; Karlsson et al., 1999, 2008; Antunes et al., 2011), temperature changes (Karlsson et al., 2003), the second messenger cyclic di-guanosyl-5′ monophosphate (c-di-GMP) (McKee et al., 2013) and quorum sensing auto-inducing peptides (Darkoh et al., 2015, 2016). The Gram-positive global transcriptional regulators CodY and CcpA are the major transcriptional regulators of the two toxins, controlling toxin gene expression by binding directly to either the *tcdR* promoter regions or the promoter region of *tcdA* and *tcdB* in response to nutrient sufficiency or carbon catabolite repression (Dineen et al., 2007, 2010; Antunes et al., 2011, 2012). The sigma factors SigH and SigD are also involved in the regulation of toxin gene expression (Saujet et al., 2011; McKee et al., 2013; Meouche et al., 2013). The toxin synthesis is also controlled by regulators involved in the control of the initiation of sporulation (Mackin et al., 2013) and the flagellar regulon (Dingle et al., 2011; Aubry et al., 2012; Baban et al., 2013).

Cysteine is an important sulfur-containing amino acid that plays a major role in cellular physiology. Cysteine is required for the biogenesis of sulfur-containing cofactors, such as biotin, lipoic acid, molybdopterin, and thiamine, as well as FeS clusters (Jez and Dey, 2013; Pace and Weerapana, 2013; Black and Dos Santos, 2015; Luebke and Giedroc, 2015). Cysteine is also found in the catalytic sites of several enzymes and involved in protein folding and assembly through the formation of disulfide bonds (Mueller, 2006; Hidese et al., 2011). Bacterial virulence has been linked to cysteine availability in several pathogenic bacteria. In *C. perfringens* and *Bordetella pertussis*, toxin synthesis is repressed in the presence of cysteine (Gooder and Gehring, 1954; Bogdan et al., 2001). In *Staphylococcus aureus*, CymR is the master regulator of cysteine metabolism and plays a major role in the regulation of virulence and adaptation to survival within the host (Soutourina et al., 2010; Ji et al., 2012). Cysteine significantly downregulates toxin synthesis in *C. difficile* strains VPI 10463 and 630Δ*erm* (Karlsson et al., 2000; Dubois et al., 2016), but the molecular mechanisms involved in the repression of toxin production by cysteine remains unclear. A recent study identified SigL as a major mediator of the cysteine-dependent regulation of toxin gene expression in *C. difficile* (Dubois et al., 2016). The inactivation of *sigL* in *C. difficile* caused the derepression of toxin gene expression in the presence of cysteine. However, no SigL-dependent promoter has been identified upstream from *tcdA, tcdB*, or *tcdR*, suggesting that SigL indirectly regulates the pathogenicity locus (PaLoc) genes. The production of H_2_S was also found strongly reduced in the *sigL* mutant compared with that in the parental strain 630Δ*erm*, suggesting that the cysteine-dependent repression of toxin production is probably attributable to the accumulation of cysteine by-products rather than to cysteine itself. Cysteine is actively catabolized by cysteine-degrading enzymes, and SigL may control cysteine degradation in *C. difficile*. Therefore, understanding the cysteine-degrading enzymes in *C. difficile* is essential.

The main enzymes involved in cysteine degradation are the pyridoxal 5′-phosphate (PLP)-dependent cysteine desulfhydrases (C-S-lyases), which are quite diverse (Auger et al., 2005; Hidese et al., 2011; Oguri et al., 2012). In *Escherichia coli*, five enzymes with C-S-lyase activity have been investigated in considerable detail: TnaA, MetC, CysK, CysM, and MalY (Awano et al., 2003, 2005). Growth phenotype and transcriptional analyses have suggested that TnaA contributes primarily to cysteine degradation *in vivo* and is induced by the addition of cysteine to the culture. CdsH appears to be the major cysteine-degrading and sulfide-producing enzyme in *Salmonella* under aerobic conditions and mutants with deletions of *cdsH* show increased sensitivity to cysteine toxicity. The *cutR* gene encodes a putative Lrp/AsnC family transcriptional regulator that mediates the regulation of *cdsH* expression by cysteine (Oguri et al., 2012). Recently, a new class of enzymes, the cysteine desulfidases, has been described in *Methanocaldococcus, Salmonella* and *Yersinia* (Tchong et al., 2005; Méndez et al., 2011; Loddeke et al., 2017). These enzymes function with Fe-S clusters and seemingly under anaerobiosis. Using zymography with cysteine as the substrate, Dubois et al. detected three bands in the crude extract of *C. difficile* strain 630Δ*erm* grown with cysteine, indicating that *C. difficile* has at least three enzymes having C-S-lyase activities and MalY/PatB corresponded to the α band (Dubois et al., 2016). However, the two other enzymes remain to be identified. In the present study, we identified the homologous cysteine desulfidase gene *cdsB* in *C. difficile* and constructed a *cdsB* mutant to examine the contribution of CdsB to the regulation of toxin synthesis and cysteine degradation in *C. difficile*.

## Materials and methods

### Bacterial strains and growth conditions

The *C. difficile* strains were cultured in BHIS, TY, or TYC (TY with 5 mM cysteine) broth in an anaerobic chamber (ShelLab, USA). The following antibiotics were added to the medium as necessary, at the indicated concentrations: d-cycloserine, cefoxitin, lincomycin, or thiamphenicol (Heap et al., 2007). All materials used for *C. difficile* culture were pre-reduced in the anaerobic chamber for more than 2 h to remove oxygen. The *E. coli* strains were grown aerobically at 37°C in Luria–Bertani medium supplemented with the appropriate antibiotics. The bacterial strains and plasmids used in this study are presented in Table 1.

**Table 1:** Strains and plasmids used in this study.

**Name**	**Description**	**Reference or origin**
**BACTERIAL STRAINS**		
*E. coli* Top10	DNA cloning and plasmid amplification	Laboratory stock
*E. coli* CA434	Conjugation donor	Laboratory stock
*C. difficile* 630Δ*erm*	Derivative of 630 strain, erythromycin sensitive strain	Laboratory stock
WT/pMTL84151	*C. difficile* 630Δ*erm* + pMTL84151	This study
*cdsB*::*ermB*/pMTL84151	*C. difficile* 630Δ*erm cdsB::ermB*+ pMTL84151	This study
*cdsB::ermB*/pMTL-*cdsB*	*C. difficile* 630Δ*erm cdsB::ermB+* pMTL-*cdsB*	This study
*sigL*::*ermB*/pMTL84151	*C. difficile* 630Δ*erm sigL::ermB*+ pMTL84151	This study
*sigL::ermB*/pMTL-*sigL*	*C. difficile* 630Δ*erm sigL::ermB+*pMTL-*sigL*	This study
WT/pRPF-P*_*cdsB*_*	*C. difficile* 630Δ*erm* + pRPF-P*_*cdsB*_*	This study
*sigL*::*ermB*/pRPF-P*_*cdsB*_*	*C. difficile* 630Δ*erm sigL::ermB*+ pRPF-P*_*cdsB*_*	This study
*cdsR*::*ermB*/pRPF-P*_*cdsB*_*	*C. difficile* 630Δ*erm cdsR*:*ermB* + pRPF-P*_*cdsB*_*	This study
WT/pRPF185	*C. difficile* 630Δ*erm* + pRPF185	This study
WT/pRPF-P*_*cdsB*_*_−223_	*C. difficile* 630Δ*erm* + pRPF-P*_*cdsB*_*_−223_	This study
WT/pRPF-P*_*cdsB*_*_−153_	*C. difficile* 630Δ*erm* + pRPF-P*_*cdsB*_*_−153_	This study
WT/pRPF-P*_*cdsB*_*_−103_	*C. difficile* 630Δ*erm* + pRPF-P*_*cdsB*_*_−103_	This study
**PLASMIDS**		
pMTL007	Clostridial ClosTron plasmid	Laboratory stock
pMTL007-*cdsB815s*	ClosTron plasmid pMTL007 containing retargeted region to *cdsB*	This study
pMTL007-*sigL454s*	ClosTron plasmid pMTL007 containing retargeted region to *sigL*	This study
pMTL007-*cdsR-523s*	ClosTron plasmid pMTL007 containing retargeted region to *cdsR*	This study
pMTL84151	Clostridia expression vector	Laboratory stock
pMTL-*cdsB*	pMTL84151 carrying the *C. difficile* 630Δ*erm cdsB* gene and its native promoter	This study
pMTL-*sigL*	pMTL84151 carrying the *C. difficile* 630Δ*erm sigL* gene and its native promoter	This study
pRPF185		Laboratory stock
pRPF-P*_*cdsB*_*	pRPF185 carrying the *native C. difficile* 630Δ*erm cdsB* promoter	This study
pRPF-P*_*cdsB*_*_−223_	pRPF185 carrying the fragment (−223 to +47 bp) of the *native C. difficile* 630Δ*erm cdsB* promoter	This study
pRPF-P*_*cdsB*_*_−153_	pRPF185 carrying the fragment (−153 to +47 bp) of the *native C. difficile* 630Δ*erm cdsB* promoter	This study
pRPF-P*_*cdsB*_*_−103_	pRPF185 carrying the fragment (−103 to +47 bp) of the *native C. difficile* 630Δ*erm cdsB* promoter	This study

### Construction of *C. difficile* mutants

The *C. difficile* mutants were generated with the method of the ClosTron gene knock-out system, as described previously (Heap et al., 2007), combined with the TargeTron® Gene Knockout System Kit (Sigma-Aldrich). The intron DNA fragments were amplified with overlap PCR using specific retargeted primers (shown in Table S1) and then cloned into the *Hin*dIII and *Bsr*GI restriction sites of pMTL007, using *E. coli* TOP10 as the host cells. After confirmation with PCR and DNA sequencing, the derived pMTL007 plasmids were each used to transform conjugative *E. coli* CA434, and then transferred into *C. difficile* 630Δ*erm* by conjugation. The *C. difficile* transconjugants were selected in the presence of thiamphenicol, erythromycin, d-cycloserine, and cefoxitin. Once colonies appeared on the plates, the mutants were verified with PCR screening using the primers shown in Table S1. Southern blotting was performed with a DIG-High Prime Labeling and Detection Kit (Roche), according to the manufacturer's instructions. For the complementation experiments, each target gene, together with its native constitutive promoter, was cloned into pMTL84151. Using *E. coli* CA434 as the donor, the complementary plasmids were transferred individually into the *C. difficile 630*Δ*erm* mutant strains to generate the strains used in this study (Table 1).

### RNA isolation and quantitative reverse transcription-PCR (qRT-PCR)

To isolate RNA, 3 ml aliquots of cultures grown in TY or TYC broth were harvested by centrifugation (4,000 × g for 10 min at 4°C). The total RNA was extracted from the cell pellets with the RNAprep pure Kit (for Cell/Bacteria) (TIANGEN). The PrimeScript RT Reagent Kit with gDNA Eraser (TaKaRa) was used to synthesize the cDNA, according to the manufacturer's instructions. The synthesized cDNA was stored at −20°C.

The relative expression levels of the target transcripts were determined with SYBR Premix Ex Taq (TaKaRa), according to the manufacturer's protocol. The primers used for real-time PCR in this study are presented in Table S1. The data were analyzed with the ΔC_T_ method as previously described (Schmittgen and Livak, 2008). The expression levels of the genes were normalized using the amplification efficiencies and the expression levels of the reference gene *rpsJ*. At least three biological replicates were assayed. The statistical analysis was performed with two-way analysis of variance (ANOVA) and a *P*-value ≤ 0.05 was considered significant.

### Zymography assay

Zymography was used to detect C-S-lyase activities as described previously (Auger et al., 2005) with some modifications. Crude extracts of the native proteins harvested from TY or TYC broth were run on a nondenaturing gel (12% polyacrylamide in Tris–glycine buffer). After electrophoresis, the gel was washed twice with 50 ml of Tris/HCl (50 mM, pH 7.4) and then incubated at 37°C for 1–4 h under anaerobic conditions in the following solution: 50 mM Tris/HCl (pH 7.4), 10 mM MgCl_2_, 0.5 mM Pb(NO_3_)_2_, 0.4 mM PLP, 5 mM dithiothreitol and 10 mM cysteine. The H_2_S formed during the enzymatic reaction precipitated as insoluble PbS, so the C-S-lyase activity was measured as the amount of precipitated PbS.

### Detection of H_2_S production

Cells were grown in TY or TYC medium for 12 h and H_2_S production was then detected with Hydrogen Sulfide Test Strips (Sigma), which turned black in the presence of H_2_S.

### Cell culture and cell cytotoxicity assay

To assay cytotoxicity, cultures of the *C. difficile* strains grown anaerobically in TY or TYC broth were centrifuged (4,000 × g for 10 min at 4°C), and filter sterilized. The filter-sterilized supernatants were then serially diluted twofold or 4-fold and added to monolayers of Vero cells preincubated in 96-well plates. Cytotoxicity was recorded after 24 h. The negative control was treated with fresh medium. The end-point titer was defined as the first dilution in the series in which the morphology of the Vero cells was the same as that of the negative control. For the neutralization assay, the diluted supernatants from 24 h cultures were incubated with appropriately diluted anti-TcdA or anti-TcdB serum for 1 h at 37°C and then added to Vero cell monolayers. Each experiment was performed three times in duplicate. The statistical analysis was performed with two-way ANOVA with multiple-comparisons test, and *P* ≤ 0.05 was considered statistically significant.

Vero cells were cultured in Dulbecco's modified Eagle's medium supplemented with 10% fetal bovine serum (Gibco) and 1% (v/v) penicillin–streptomycin at 37°C in a humidified atmosphere containing 5% CO_2_. For the cytotoxicity assay, Vero cells were seeded into 96-well plates at densities of approximately 5 × 10^4^ cells/ml and incubated for 20–24 h before the assay.

### Dot blotting analysis

The supernatants from *C. difficile* cultures grown anaerobically in TY broth were concentrated with 10-kDa Amicon® Ultra-4 Centrifugal Filter Units (Merck Millipore), and the cell densities were standardized. The samples were spotted onto a nitrocellulose membrane and air-dried. The membranes were blocked with 5% nonfat dry milk in phosphate-buffered saline (PBS) containing 0.05% Tween 20 for 1 h, and incubated with mouse anti-TcdA serum (maintained in our laboratory) and then with a horseradish-peroxidase conjugated anti-mouse secondary antibody. The immunological spots were visualized with the SuperSignal West Pico Chemiluminescent Substrate Kit (Thermo Scientific), according to the manufacturer's instructions.

### 5′-RACE analysis

The extraction and purification of total RNA were performed as described above. Transcriptional start site (TSS) of *cdsB* was determined using the SMARTer™ RACE cDNA Amplification Kit (Clontech) according to manufacturer instructions. Gene-specific primers and universal primer mix were used to amplify the 5′ end of *cdsB* mRNA (Table S1).

### Construction of *cdsB* promoters with *gusA* gene fusions

To generate the *cdsB* promoter-reporter gene fusions, regions of various lengths upstream of the *cdsB* promoters were PCR-amplified from strain *630*Δ*erm* genomic DNA with primers listed in Table S1. These products were independently ligated into the *Kpn*I/*Sac*I sites of pRPF185, which contains a *C. difficile* codon-optimized *gusA* gene (Fagan and Fairweather, 2011), to generate the plasmids listed in Table 1. The recombinant plasmids were confirmed by sequencing and then transferred into *630*Δ*erm*, the *sigL*::*ermB* or the *cdsR*::*ermB* strains, which were selected by resistance to thiamphenicol and verified by PCR.

### β-Glucuronidase assay

The *C. difficile* strains containing the promoter–reporter gene fusions listed in Table 1 were grown to late exponential phase (OD_600_, 1.5), and the cells were harvested and stored at −20°C. The samples were then lysed and analyzed for β-glucuronidase activity, as previously described (Fagan and Fairweather, 2011). Briefly, the pellets stored at −20°C were resuspended in 0.5 ml of PBS and incubated at 37°C for 40 min to generate whole-cell lysates. The enzymatic reaction was initiated by the addition of 100 μl of a 6 mM solution of p-nitrophenyl-β-d-glucuronide. After incubation at 37°C, the reactions were stopped by the addition of 240 μl of 1 M Na_2_CO_3_. The tubes were centrifuged at 12,000 × g for 5 min to remove the cell debris and OD_405_ was measured with a MD SpectraMax M5 spectrophotometer. Specific activity was calculated with the formula: (OD_405_ × 1,000)/(OD_600_ × t [min] × 1.25 × volume [ml]). The results are presented as the means of the calculated activity and the standard errors of the means of at least three replicates. The data were analyzed with two-way ANOVA with Dunnett's multiple-comparisons test.

## Results

### *cdsB* homolog (CD630_32320) found in the *C. difficile* 630 genome

**Figure 1:**
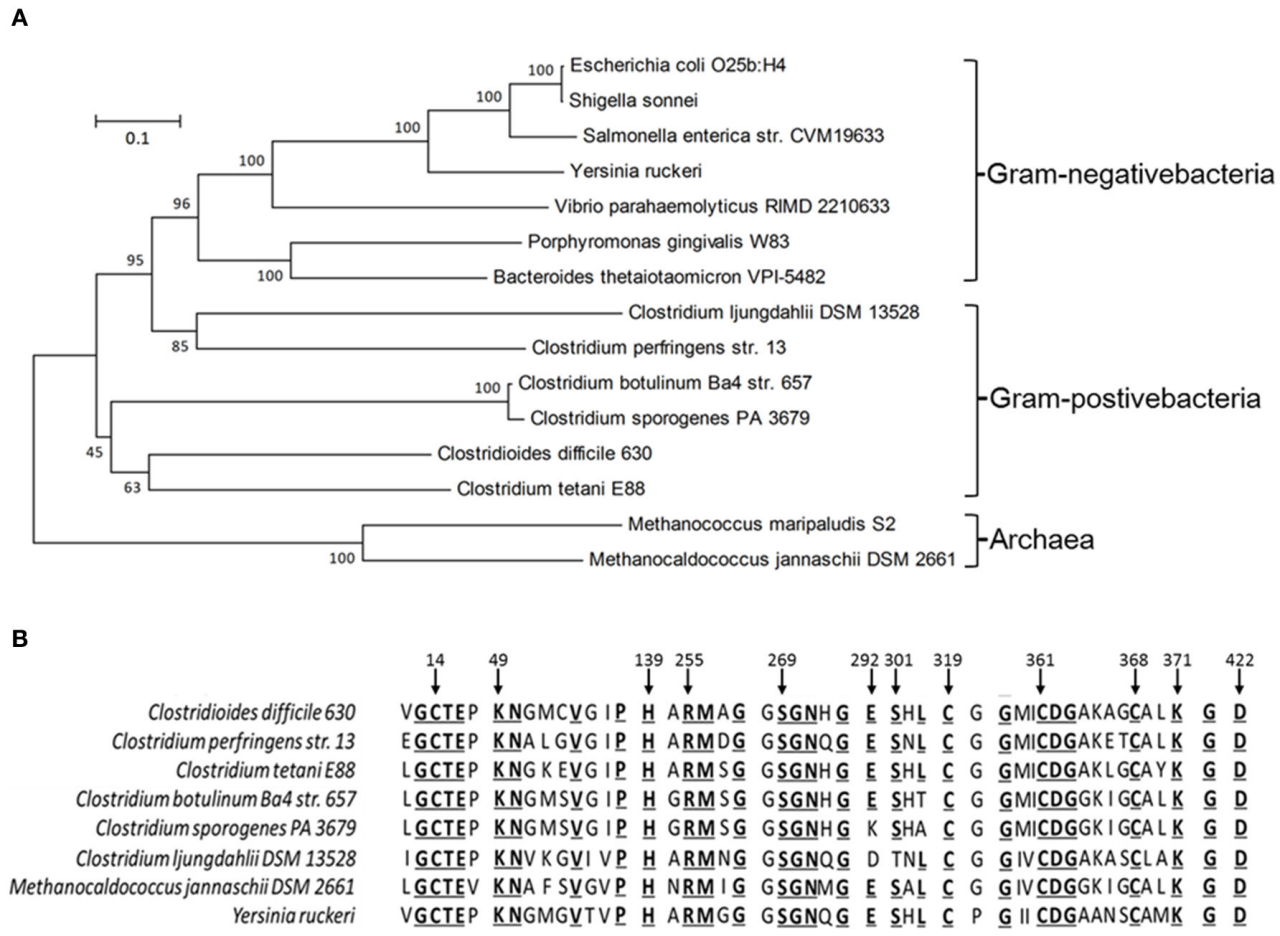
Phylogenetic tree **(A)** and conserved residues **(B)** deduced from the CdsB protein sequence, showing the relationships between *C. difficile* and other bacteria. GenBank accession numbers are indicated: *E. coli* O25b:H4 (ANK03584.1), *Shigella sonnei* (WP_000460525.1), *S. enterica str*. CVM19633 (ACF92064.1), *Y. ruckeri* (ADO66727.1), *Vibrio parahaemolyticus* RIMD 2210633 (NP_798552.1), *Porphyromonas gingivalis* W83 (AAQ66048.1), *Bacteroides thetaiotaomicron* VPI-5482 (NP_810993.1), *C. ljungdahlii* DSM 13528 (OAA87710.1), *C. perfringens* str. 13 (BAB80512.1), *C. botulinum* Ba4 str. 657 (ACQ52197.1), *C. sporogenes* PA 3679 (EHN13584.1), *C. difficile* 630 (YP_001089749.1), *C. tetani* E88 (AAO36786.1), *M. maripaludis* S2 (CAF31024.1), *M. jannaschii* DSM 2661 (AAB99029.1).

A cysteine desulfidase, CdsB, has been described as catalyzing the hydrolysis of cysteine to sulfide, ammonia, and pyruvate in *M. jannaschii* (Tchong et al., 2005). When we searched the genome of *C. difficile* 630, we identified the *cdsB* homologous gene CD630_32320. A phylogenetic analysis was performed with the MEGA software using the deduced amino acid sequences of the CdsB proteins derived from the genomes of *C. difficile* 630 and 14 other bacterial strains. This showed that the CdsB protein is conserved in many facultatively anaerobic and anaerobic bacteria, but not in aerobic bacteria, and that it has evolved into two clearly demarcated branches, the enbacterial branch and the archeal branch (Figure 1A). CdsB homologs were also found widely distributed in the genomes of the genus *Clostridium*. Tchong et al. identified four conserved cysteine residues (C25, C282, C322, and C329) in the CdsB protein in *M. jannaschii*. Three of them (C282, C322, and C329) are ligands for the [4Fe–4S] center, and the thiolate of Cys25 acts as a base to abstract the R-hydrogen in the first step of elimination (Tchong et al., 2005). Based on a multiple-sequence alignment of the CdsB proteins using ClustalW, the CdsB protein encoded in *C. difficile* contains all four cysteine residues that are conserved in specific positions of the l-cysteine desulfidase of *M. jannaschii* (Figure 1B). As well as the conserved cysteine residues, an additional 25 amino acids reported in the CdsB protein of *M. jannaschii* are conserved in *C. difficile*. Among them, nine amino acid residues (K49, H139, R255, S269, E292, S301, D362, K371, and D422) that are considered to be involved in the catalytic mechanism or substrate binding in *M. jannaschii* CdsB also occur in *C. difficile* CdsB (Figure 1B). On the basis of these conserved amino acids and the phylogenetic analysis results shown above, we speculated that CdsB has similar functions in *C. difficile* as in *M. jannaschii* and other organisms, and is probably a cysteine desulfidase involved in bacterial virulence.

### Effect of excess cysteine on the transcription of *cdsB* in *C. difficile* 630Δ*erm*

**Figure 2:**
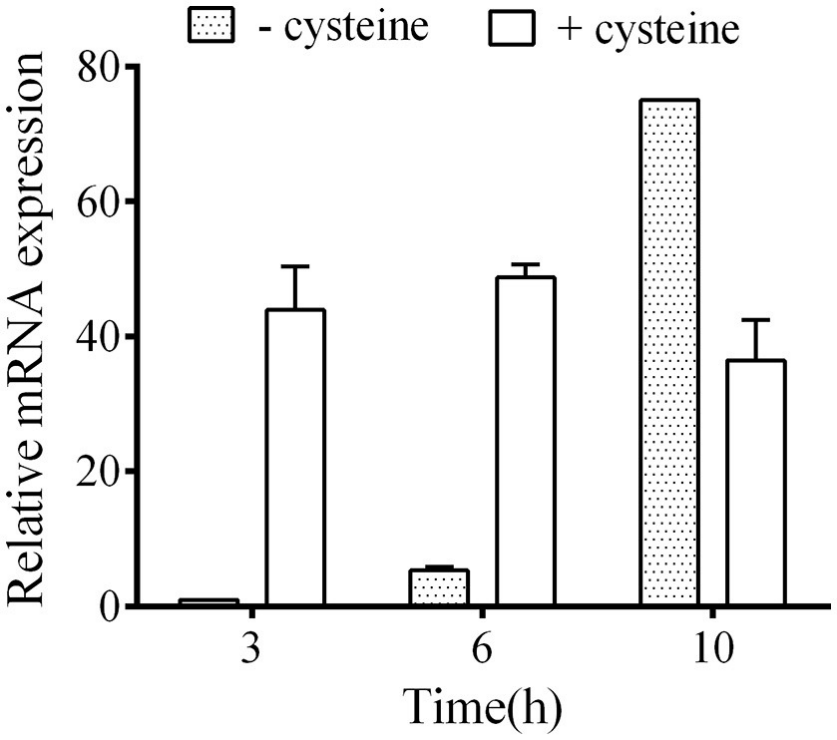
Quantitative RT–PCR analysis of *cdsB* transcription during *C. difficile* growth in TY or TYC broth. Results show the relative expression of *cdsB* normalized to that of the *rpsJ* gene. Error bars correspond to the standard deviations of three biological replicates.

To explore the role of CdsB in *C. difficile* growth and toxin production during supplementation with cysteine, the expression of *cdsB* was first analyzed with qRT-PCR during different growth phases of *C. difficile* 630Δ*erm* in TY or TYC broth (Figure 2). In TY broth, the *cdsB* gene was mainly expressed during the late exponential phase (10 h) and its expression during the early and mid- exponential phases (3 and 6 h, respectively) was lower. The trend in the transcription of *cdsB* was quite different when the cells were cultured with cysteine. The *cdsB* expression was much higher than in cells grown without cysteine during both the early and mid-exponential phases, but decreased during the late exponential phases of growth. These results indicate that transcription of *cdsB* is induced early in growth by the presence of cysteine. The reduced expression of *cdsB* at the end of the exponential growth phase in TYC might be attributable to the degradation of cysteine and its consequent depletion.

### C-S-lyase activity present in *C. difficile cdsB* mutant

To explore the role of CdsB in *C. difficile*, we inactivated the *cdsB* gene in *C. difficile* 630Δ*erm* with the ClosTron technology. The insertion of a group II intron into the *cdsB* gene (insertion site at 815s) was confirmed with PCR amplification and Southern blotting (Figure S1). To generate the complemented strain, the vector pMTL84151-*cdsB* was transferred into strain *C. difficile 630*Δ*erm cdsB::ermB* to produce strain *cdsB*::*ermB*/pMTL-*cdsB*. To exclude the effects of the heterologous plasmid on the strains, the pMTL84151 vector was transferred into strains *C. difficile* 630Δ*erm* and *C. difficile 630*Δ*erm cdsB*::*ermB* to generate the parental strain WT/pMTL84151 and strain *cdsB*::*ermB*/pMTL84151.

**Figure 3:**
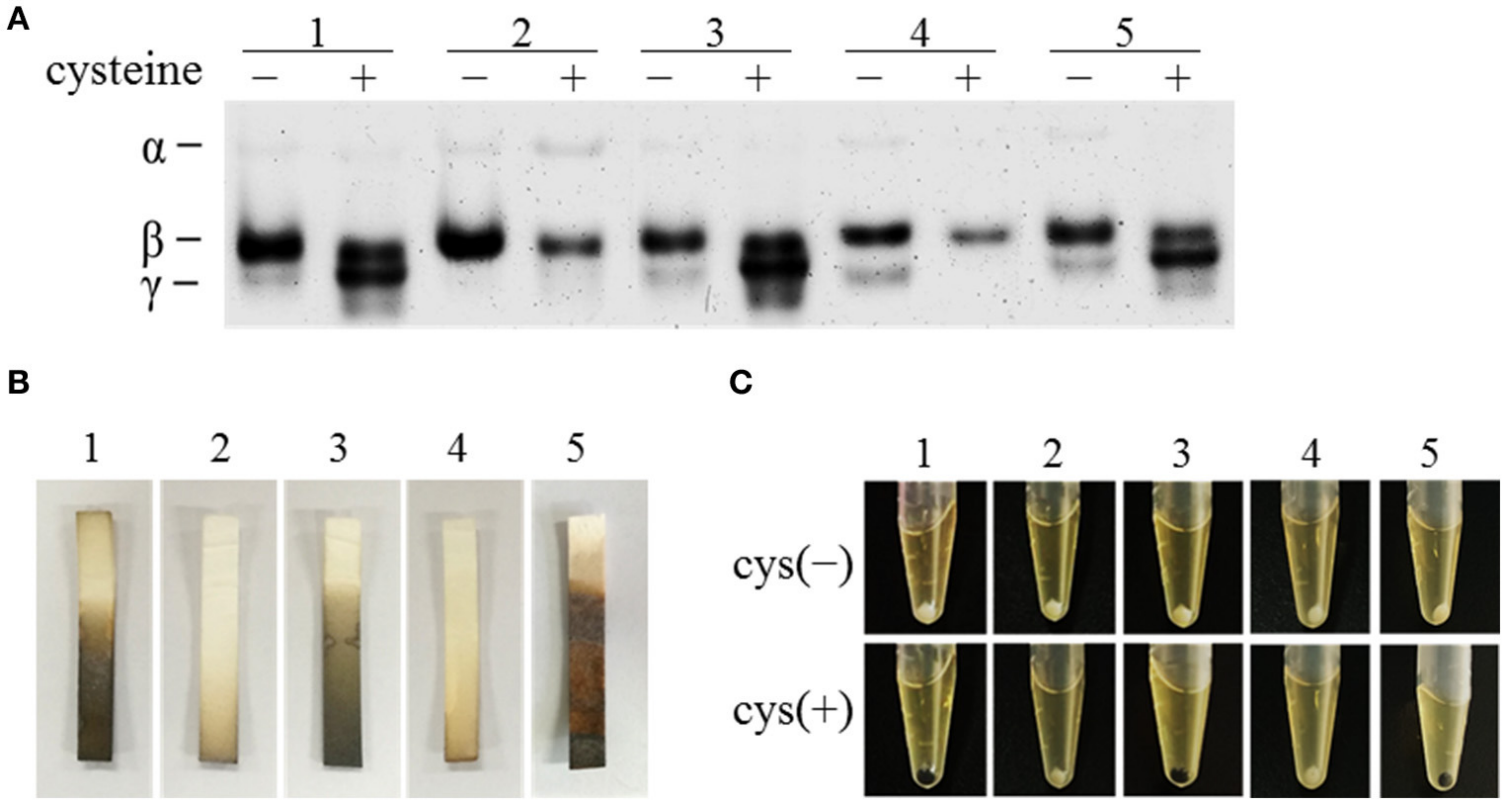
Effect of *cdsB* inactivation on cysteine degradation and H_2_S production in *C. difficile* 630Δ*erm*. **(A)** Detection of C-S-lyase activities with zymography. The strains were grown in TY broth (−) or TYC broth (+). **(B)** Detection of H_2_S production with Hydrogen Sulfide Test Strips. The strains were grown in TYC broth for 12 h. **(C)** Colors of bacterial pellets grown in TY or TYC broth for 12 h. WT/pMTL84151 (lane 1), *cdsB*::*ermB*/pMTL84151 (lane 2), *cdsB::ermB*/pMTL-*cdsB* (lane 3), *sigL*::*ermB*/pMTL84151 (lane 4), and *sigL::ermB*/pMTL-*sigL* (lane 5).

We performed zymography to compare the C-S-lyase activities in the *C. difficile* strains and to investigate the role of CdsB in cysteine degradation in *C. difficile*. The C-S-lyase activities in crude extracts of the parental WT/pMTL84151 strain, the *cdsB::ermB*/pMTL84151 strain, and the *cdsB::ermB*/ pMTL-*cdsB* strain cultured in TY or TYC broth were monitored directly on a native gel with incubation in the presence of cysteine and Pb(NO_3_)_2_. Three bands (α, β, and γ) were detected in the crude extracts of the WT/pMTL84151 strain, which is consistent with a previous study (Dubois et al., 2016). The intensity of the γ band was clearly increased in the presence of cysteine (Figure 3A, lane 1). However, unlike the parental WT/pMTL84151 strain, the γ band was absent from crude extracts of the *cdsB::ermB*/pMTL84151 strain grown in either TY or TYC broth, whereas the γ band reappeared in the complemented strain *cdsB::ermB*/pMTL-*cdsB* (Figure 3A, lanes 2 and 3). This result suggests that the enzyme in the γ band was a CdsB, and was a cysteine-induced enzyme, clearly induced in the presence of cysteine. Interestingly, the expression of the CdsB protein corresponding to the γ band was strongly reduced in the *sigL::ermB/* pMTL84151 strain and restored in the *sigL::ermB*/pMTL-*sigL* strain, suggesting that the expression of CdsB is regulated by SigL. The expression of the enzyme in the β band was relatively steady, with or without the addition of cysteine, suggesting that it is a constitutively expressed cysteine-independent enzyme involved in cysteine degradation in *C. difficile*. A previous study indicated that the enzyme corresponding to α band is MalY-dependent, and that in the presence of cysteine, the expression of MalY was reduced in the *sigL* mutant (Dubois et al., 2016). In the present study, all the strains expressed a small amount of protein corresponding to α band when cultured without cysteine, which probably reflected the constitutive expression of MalY. However, when cultured with cysteine, the expression of MalY decreased in strain *sigL::ermB/*pMTL84151, which is consistent with a previous study (Dubois et al., 2016), suggesting that expression of malY is possibly regulated by SigL. The expression of MalY in strain *cdsB*::*ermB*/pMTL84151 increased, which is probably due to the disruption of a major cysteine-inducible cysteine desulfidase gene (*cdsB*) and the expression of other proteins that with C-S-lyase activities may be affected by the presence of cysteine. According to these results, the expression of MalY might be affected by cysteine, SigL, and CdsB, the regulatory mechanisms for this phenomenon remain to be identified.

We also showed that the production of H_2_S as a result of cysteine degradation was strongly reduced in the *cdsB::ermB*/pMTL84151 strain, compared with that in the parental WT/pMTL84151 strain, but was restored in the complemented *cdsB::ermB*/pMTL-*cdsB* strain (Figure 3B). The H_2_S production by the *sigL::ermB/*pMTL84151 strain and *sigL::ermB*/pMTL-*sigL* strain, detected with H_2_S test strips, was consistent with the results of a previous study (Dubois et al., 2016), which showed that the inactivation of *sigL* strongly reduced the amount of H_2_S generated in the presence of cysteine. It has been reported that cells of the strain 630Δ*erm* turn black when grown in TYC broth (Dubois et al., 2016), which we also observed in the parental WT/pMTL84151 strain, the complemented *cdsB::ermB*/pMTL-*cdsB* strain, and the complemented *sigL::ermB*/pMTL-*sigL* strain, but not in the *cdsB::ermB/*pMTL84151 strain or the *sigL::ermB/*pMTL84151 strain (Figure 3C). The black deposit in the anaerobic waste collection system was probably an iron-sulfide precipitate resulting from the production of high levels of H_2_S during cysteine degradation (Nielsen et al., 2008). Therefore, the inactivation of the *cdsB* gene in *C. difficile* results not only in changes in the expression of the active C-S-lyases, but also in lower H_2_S production in the presence of cysteine. This suggests that CdsB in *C. difficile* is an important cysteine-inducible cysteine-degrading enzyme.

### Inactivation of *cdsB* gene in *C. difficile* 630Δ*erm* relieves cysteine-dependent repression of toxin synthesis

**Figure 4:**
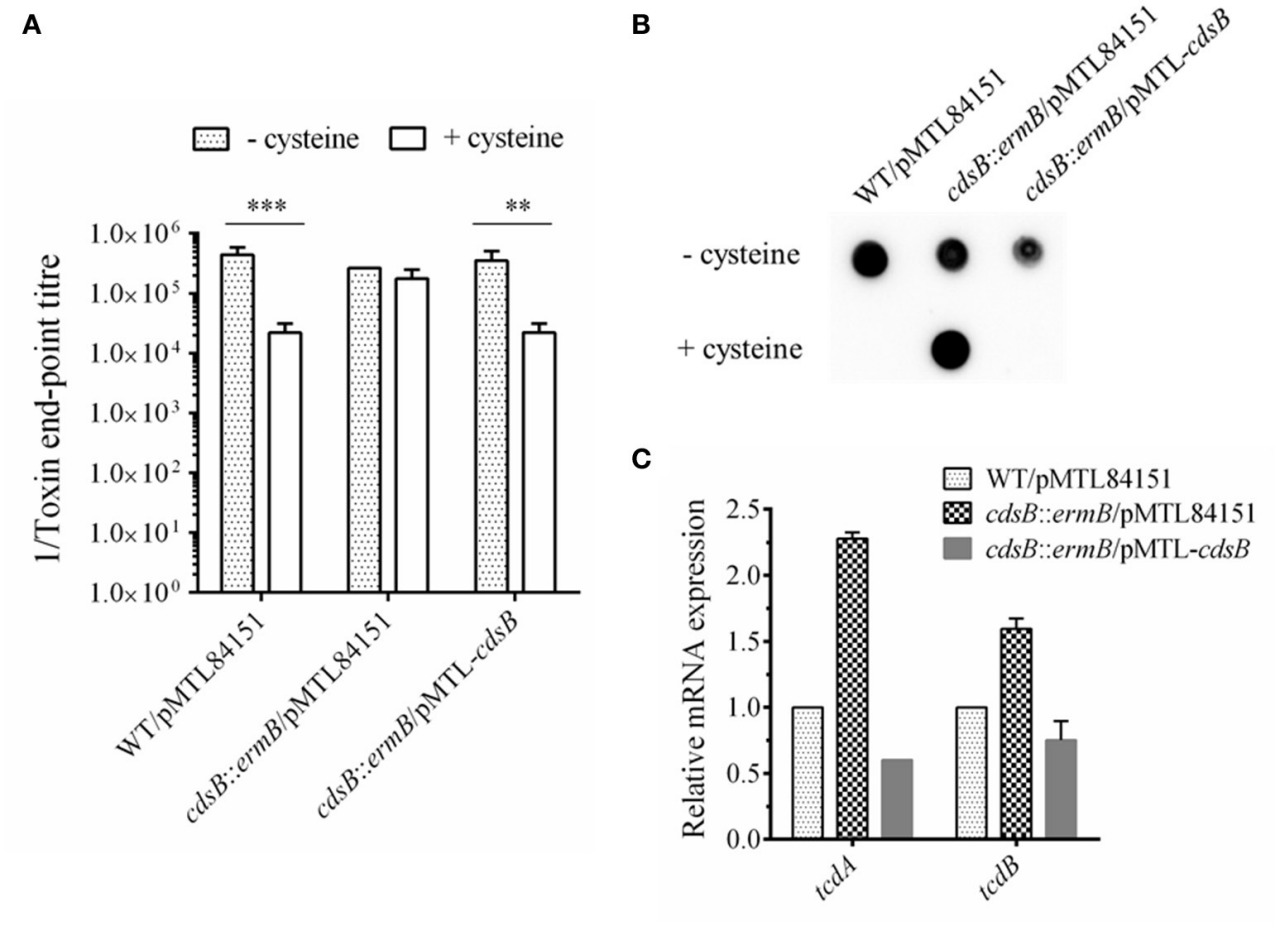
Effect of *cdsB* inactivation on toxin production in *C. difficile* 630Δ*erm*. **(A)** Cytotoxicity analysis on Vero cells. Supernatants obtained from *C. difficile* strains cultured with or without cysteine for 48 h were serially diluted 4-fold and added to monolayers of Vero cells preincubated in 96-well plates. Cytotoxicity was recorded after 24 h. The end-point titer was defined as the first dilution in the series in which the morphology of the Vero cells was the same as that of the negative control. Negative control was treated with fresh medium. ^**^P < 0.01; ^***^P < 0.001. **(B)** TcdA dot blotting analysis of toxin levels in the supernatants of WT/pMTL84151 (lane 1), *cdsB*::*ermB*/pMTL84151 (lane 2), and *cdsB::ermB*/pMTL-*cdsB* (lane 3) cultured for 48 h with or without cysteine. **(C)** Quantitative RT–PCR analysis of *tcdA* and *tcdB* transcription in the presence of cysteine. The *rpsJ* gene was used as the reference gene. Error bars correspond to the standard deviations of three replicates.

The toxin levels in the supernatant of strain *cdsB::ermB/*pMTL84151 were compared with those in the parental WT/pMTL84151 with cell cytotoxicity assay. The results are presented in Figure 4A and Figure S2. In the parental WT/pMTL84151 strain, the toxin levels were significantly lower in TYC broth than in TY broth. However, in the *cdsB::ermB/*pMTL84151 strain, no obvious difference in toxin levels was detected in TY and TYC broth. The level of toxin production in the complemented *cdsB::ermB*/pMTL-*cdsB* strain was similar to that in the parental WT/pMTL84151 strain and was repressed by the addition of cysteine. TcdA in the culture supernatants of these strains were also analyzed with dot blotting. As shown in Figure 4B, TcdA in the supernatants of the parental WT/pMTL84151 strain and the complemented *cdsB::ermB*/pMTL-*cdsB* strain disappeared in TYC broth, whereas that in the supernatant of strain *cdsB::ermB/*pMTL84151 was similar regardless of whether it was grown in TYC or TY broth. The mRNA levels of *tcdA* and *tcdB* in the *C. difficile* strains cultured in the presence of cysteine were detected with qRT-PCR, as shown in Figure 4C. The transcription levels of both *tcdA* and *tcdB* were higher in strain *cdsB::ermB/*pMTL84151 than in the parental or complemented strain. These results clearly demonstrate that the repression of toxin synthesis by cysteine was abolished in strain *cdsB::ermB/*pMTL84151, indicating that the cysteine-dependent repression of toxin expression is closely related to the cysteine degradation.

### Inhibition of the toxin expression by Na_2_S supplementation in *C. difficile* strains

**Figure 5:**
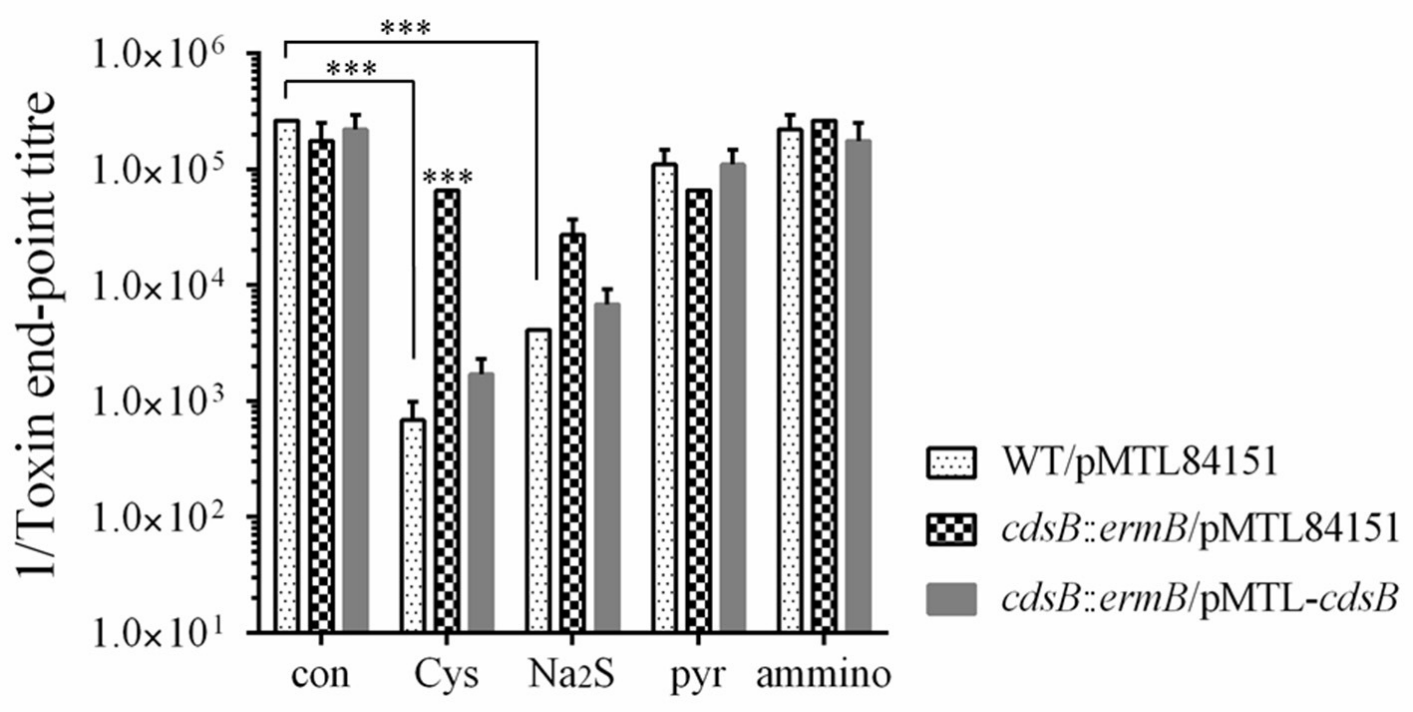
Effect of cysteine by-products supplementation on toxin production in *C. difficile* strains. Cytotoxicity analysis of toxin levels in the supernatants of WT/pMTL84151, *cdsB*::*ermB*/pMTL84151, and *cdsB::ermB*/pMTL-*cdsB* cultured for 48 h in TY broth with 10 mM cysteine, Na_2_S, pyruvate or NH_4_Cl. ^***^P < 0.001.

As the cysteine-dependent repression of toxin expression is closely related to the cysteine degradation, which is similar to a previous study's view that it is the accumulation of cysteine by-products rather than the cysteine itself that involved in the repression of toxin expression (Dubois et al., 2016), we also analyzed the toxin expression in the strains grown in TY broth supplemented with either 10 mM cysteine or 10 mM cysteine by-product (Na_2_S, pyruvate, or NH_4_Cl). As shown in Figure 5, the toxin level with the addition of Na_2_S, as well as with the addition of cysteine, was significantly reduced in the strain WT/pMTL84151. However, there were no significant differences on the toxin levels between the WT/pMTL84151 strain, the *cdsB::ermB/*pMTL84151 strain, and the *cdsB::ermB*/pMTL-*cdsB* strain in the presence of Na_2_S, which suggested that the inactivation of *cdsB* couldn't abolish the Na_2_S-dependent repression of toxin synthesis. A previous study showed that the transcripts of the *tcdA* and *tcdB* genes were downregulated by pyruvate in 630Δ*erm*, and suggested that the effect of pyruvate on toxin gene expression was partly mediated by the two-component system CD2602–CD2601 (Dubois et al., 2016). In this study, the toxin levels detected at the protein level were also slightly reduced in the strain WT/pMTL84151 in response to pyruvate. The toxin levels in the presence of pyruvate showed no significant differences among the three strains, suggesting that *cdsB* gene is irrelevant to the repression of toxin production by pyruvate. The addition of NH_4_Cl in the medium has no effects on toxin levels of the three strains. These results indicate that similar to cysteine, excess Na_2_S can significantly repress toxin expression in *C. difficile*, but the repression cannot be abolished by the inactivation of *cdsB*, which provide a reasonable proof for the view that the cysteine-dependent toxin repression is probably related to the accumulation of cysteine by-products (Dubois et al., 2016).

### Effect of *cdsB* disruption on *C. difficile* growth in response to cysteine

**Figure 6:**
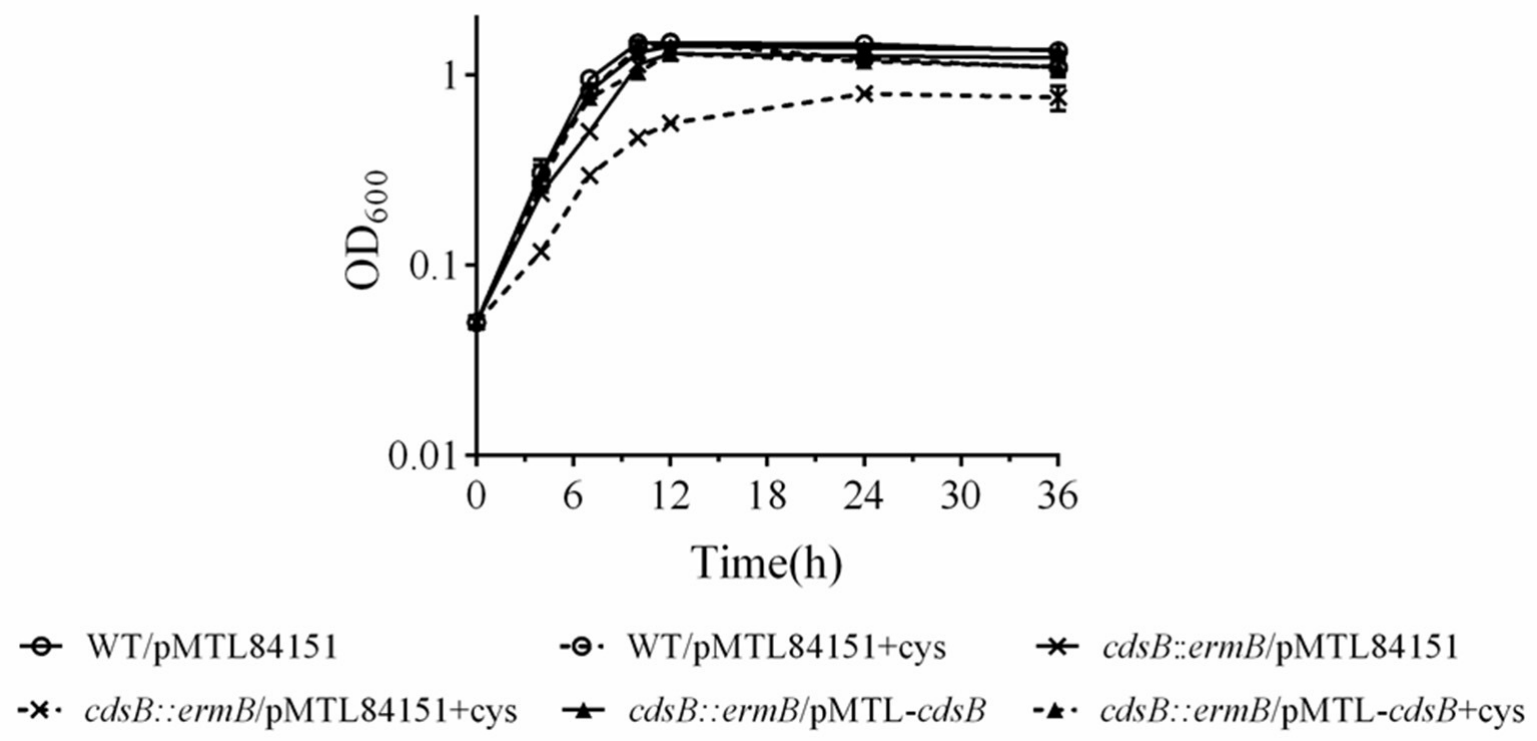
Effect of cysteine on the growth of *C. difficile* parental and *cd*sB-mutant strains. Strains were diluted to an OD_600_ of 0.05 in TY broth (solid lines) or TY broth supplemented with 0.5 mM cysteine (dotted lines). All strains grew similarly in TY alone, as shown by the solid line. ◦, WT/pMTL84151; ×, *cdsB::ermB*/pMTL84151; and ▴, *cdsB::ermB*/pMTL-*cdsB*. Growth curves were determined with three independent experiments.

To assess the effect of *cdsB* disruption on *C. difficile* growth, growth curves of these strains in TY and TYC broth were constructed based on their OD_600_ values. Single colonies of the strains were picked and cultured in BHIS broth for 16 h, and then subcultured in TY broth or TYC broth. The growth of the *cdsB::ermB/*pMTL84151 strain was similar to that of the parental strain in TY broth. However, when supplemented with 5 mM cysteine, the *cdsB::ermB/*pMTL84151 strain showed a growth defect relative to the parental strain or the *cdsB::ermB*/pMTL-*cdsB* strain (Figure 6). No obvious difference in growth was observed between the parental WT/pMTL84151 strain and the *cdsB::ermB*/pMTL-*cdsB* strain when the cells were cultured in TY or TYC broth. These results indicate that the inactivation of *cdsB* increased the sensitivity of *C. difficile* to cysteine.

### Transcriptional control of *cdsB* gene by SigL in the presence of cysteine

**Figure 7:**
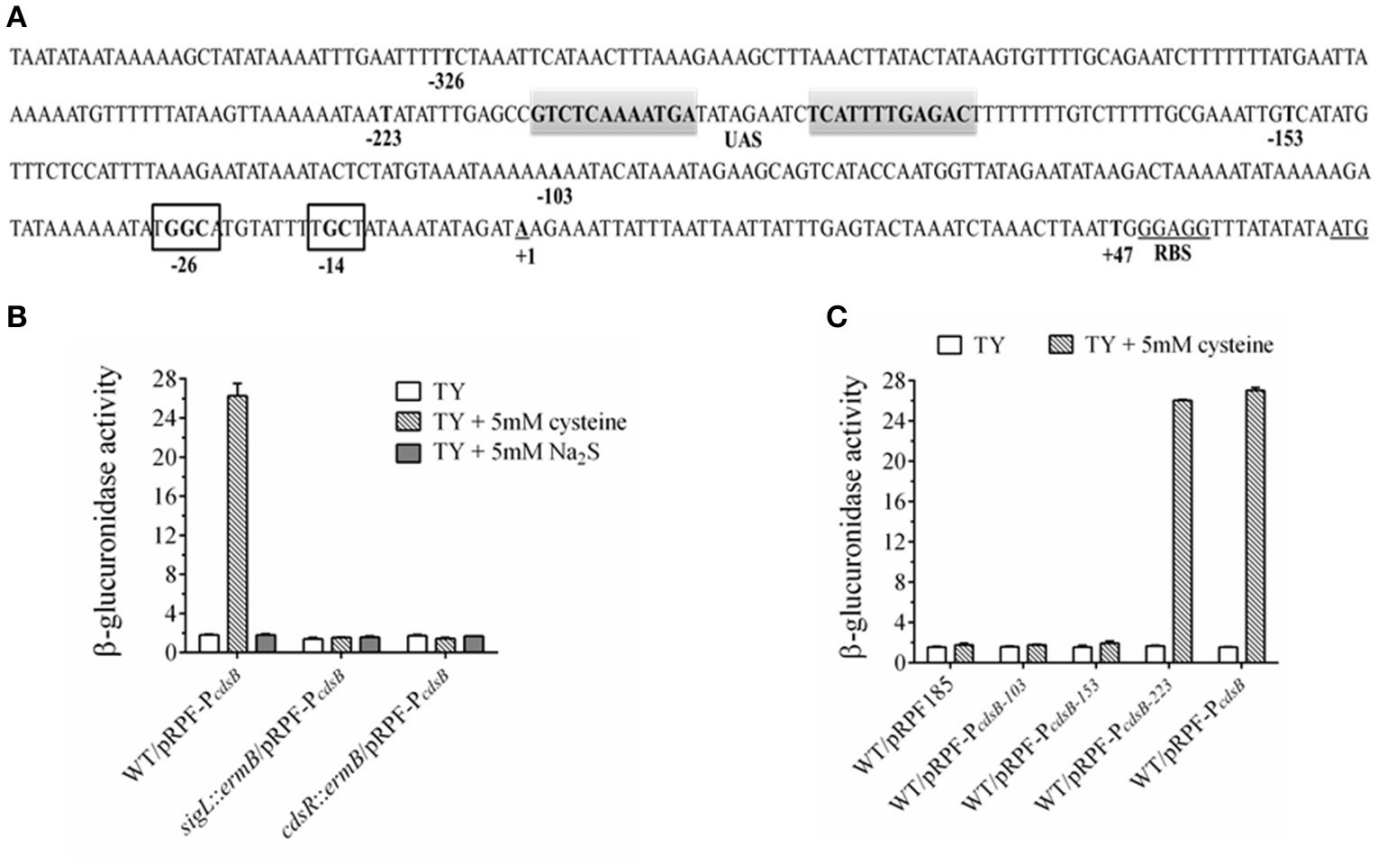
Regulation of *cdsB* expression by δ^54^ in *C. difficile*. **(A)** Promoter elements predicted to bind δ^54^ in noncoding region of the *cdsB* gene. The transcriptional start sites identified by 5′ RACE analysis are bold, underlined and identified by +1 while the ATG initiation codon and RBS is bold and underlined. The putative inverted repeat UAS is in gray shaded area while the conserved −24/−12 sequence are shown in black box. Promoter fusions of the indicated sizes marked by bold nucleotides were created. **(B)** β-Glucuronidase activity analysis to measure the *cdsB* expression in *C. difficile* strains 630Δ*erm, sigL::ermB*, and *cdsR::ermB*. Error bars represent the standard errors of the means. **(C)** β-Glucuronidase activity analysis of different fusions to identify the elements necessary for *cdsB* expression in *C. difficile*.

Based on the results described above and in a previous report (Dubois et al., 2016), we inferred that the *cdsB* gene is probably controlled by δ^54^. We searched upstream from the *cdsB* gene and found a consensus sequence of promoters recognized by δ^54^ (TTGGCACG-N4-TTGC) (Nie et al., 2015) in the region upstream from the start codon of the *cdsB* gene (Figure 7A). To confirm this, a recombinant plasmid consisting of the *cdsB* promoter fused to the *gusA* reporter gene was constructed and the β-glucuronidase activities were measured in *C. difficile* strains. The activities of the reporter fusions were assessed in the *C. difficile* 630Δ*erm* strain and the *sigL*::*ermB* strain (Figure 7B). As predicted, this region contained the promoter elements necessary to support transcription, as indicated by the β-glucuronidase activity expressed. In *C. difficile* 630Δ*erm*, higher β-glucuronidase activity was observed in the presence of cysteine than without cysteine, which is consistent with the zymographic results and qPCR described above, indicating that CdsB expression is induced by cysteine. The activity of the *cdsB* promoter was significantly reduced in the *sigL::ermB* strain cultured with TYC, which suggests that δ^54^ is required for cysteine-dependent expression from the *cdsB* promoter. The effect of Na_2_S addition on the β-glucuronidase activity was also compared between the strains (Figure 7B), and the results indicated that it was the cysteine but not cysteine by-products that induced the expression of *cdsB*. To determine the TSSs of *cdsB*, 5′-RACE analysis was further performed as described in the materials and methods. An A residue located 63 bp upstream from the *cdsB* start codon was identified (Figure 7A). Therefore, these results strongly suggested that *cdsB* is transcribed by δ^54^ associated with the core enzyme of the RNA-polymerase.

Unlike δ^70^, the δ^54^-RNA polymerase forms a stable closed complex at the promoter site, and the conversion to an open complex absolutely requires the input of ATP from an associated activator (enhancer binding proteins, EBP^54^s), which binds to the upstream activating sequence (UAS) remotely upstream from the transcription start site (Francke et al., 2011). These EBP^54^s usually consist of an N-terminal signal-recognition domain, a central activator domain, which provides the ATPase activity, and a C-terminal DNA-binding domain, and are often encoded adjacent to the controlled genes (Francke et al., 2011). Interestingly, the *CD630_32330* gene (renamed as *cdsR* in this study) upstream from the *cdsB* gene encodes a transcriptional regulator of 591 amino acids, which contains PAS, AAA-type ATPase, and DNA-binding Fis domains, and shares strong similarities with the EBP^54^s. The highly conserved “GAFTGA” amino acid sequence, which is essential for the interaction between δ^54^ and the EBP^54^ activators (Rappas et al., 2005), is also found in the CdsR amino acid sequence. To test whether CdsR is the EBP^54^ for *cdsB* expression, the activity of the *cdsB* promoter fused to the *gusA* reporter gene was assessed in the *cdsR*::*ermB* strain. Compared with the strain 630Δ*erm*, the reporter expression in this strain was significantly reduced when cultured in TYC (Figure 7B), suggesting that the *cdsR* gene is also required for the expression of *cdsB* in *C. difficile*.

The EBP^54^s usually bind UASs located between 100 and 200 bp upstream from the TSS. Sequence analyses of the region revealed two inverted repeat sequences spanning from nt −179 to −190 and nt −200 to −211, suggesting that these areas might be the UAS site for EBP^54^ and involved in regulation (Figure 7A). We created several fusions to determine the necessary sequence required for the transcription of *cdsB* (Table 1). Compared with the control plasmid pRPF185, the plasmids containing the reporter fusions with the 153-bp upstream region from the TSS of *cdsB* gene did not generate significant β-glucuronidase activity in strain 630Δ*erm* in the presence of cysteine (Figure 7C). However, the pRPF-P_*cdsB*__−223_ fusion was inducible and had strong activity in the presence of cysteine, demonstrating that the 223 bp upstream region from the TSS of *cdsB* gene is sufficient for its transcription. The region between 153 and 223 bp upstream from the *cdsB* TSS containing the putative UAS site for EBP^54^ is the necessary element for the transcription of *cdsB*. Together, these results strongly suggested that the CdsR regulator which is probably the EBP^54^ of *cdsB* is required in the transcription of *cdsB*, and that the region between 153 and 223 bp upstream from the TSS of *cdsB* gene containing the putative UAS site for EBP^54^ is necessary for its transcription.

## Discussion

A *cdsB* homolog has been found in many facultative anaerobes and anaerobes. However, the function of the encoded protein has only been examined in *M. jannaschii* and *Y. ruckeri*. A recent study reported that CyuA in *S. enterica* and *E. coli* is an anaerobic cysteine desulfidase, which possibly contributes to the coordination of sulfur assimilation and amino acid synthesis (Loddeke et al., 2017). CyuA homologs was also present in two of the most ancient organisms, archaeal methanogens and eubacterial acetogens, which suggests that CyuA/CdsB homologs exists in the universal common ancestor, before the divergence of the Archaea and Bacteria (Loddeke et al., 2017). In the present study, we identified CdsB present in *C. difficile* and in other *Clostridium* species, such as *C. perfringens, C. botulinum, C. sporogenes*, and *C. ljungdahlii*. Most of the conserved residues, including the four conserved cysteine residues, identified in *M. jannaschii* (Tchong et al., 2005) are also present in these bacteria. We infer that the CdsB homologs in these bacteria have similar functions in cysteine degradation to that of the homologs in *C. difficile* and *M. jannaschii*.

When *C. difficile* was cultured with cysteine, the *cdsB* gene was rapidly induced and expressed, and the protein catalyzed the conversion of cysteine into a large amount of H_2_S. A similar study showed that the *cdsAB* promoter was most strongly induced in the presence of cysteine in *Y. ruckeri* (Brouwer et al., 2011). The inactivation of *cdsB* in *C. difficile* altered the composition of the intracellular cysteine-degrading enzymes, and significantly reduced the accumulation of H_2_S. We clearly demonstrated that the enzyme present in the γ band was generated in the presence of cysteine, and that CdsB was the enzyme present in the inducible γ band. Dubois et al. used zymography to identify MalY as the α band. The inactivation of the *cysK* gene had no effect on the C-S-lyase activity of *C. difficile* in the presence of cysteine, suggesting that CysK is not a major C-S-lyase (Dubois et al., 2016). The enzyme corresponding to the β band seems to be a constitutive enzyme, and remains to be identified.

It has been reported that cysteine catabolism or C-S-lyase affects diverse functions, including oxidative stress, swarming motility, and virulence, in several organisms (Ji et al., 2012; Oguri et al., 2012; Luebke and Giedroc, 2015). In *Y. ruckeri*, the *cdsAB* operon is reportedly necessary for full virulence, and therefore for the development of the infectious process, based on the results of competitive inhibition and median lethal dose (LD_50_) experiments *in vivo* (Méndez et al., 2011). Dubois et al. have previously shown that SigL is involved in the regulation of cysteine-dependent repression of toxin expression and proposed that cysteine-dependent repression of toxin production is probably mainly due to the accumulation of cysteine by-products during growth (Dubois et al., 2016). In the present study, we found that the inactivation of *cdsB*, which encodes the major cysteine-inducible cysteine desulfidase in *C. difficile*, abolished the cysteine-dependent repression of toxin production, which was able to be recovered in the complemented strain containing the *cdsB* gene. These results support that the cysteine-dependent repression of toxin production is closely related to cysteine degradation. The effects of cysteine by-products on toxin synthesis were further determined, and the results showed that similar to cysteine, Na_2_S addition could also cause significant repression in *C. difficile*. Combining with the results of H_2_S production in both parental WT/pMTL84151 strain and the *cdsB::ermB/*pMTL84151 strain when cultured with cysteine, we speculate that the H_2_S accumulation might be the major cause for the repression of toxin production. Indeed, with the Na_2_S addition, the toxin level of the *cdsB::ermB/*pMTL84151 strain showed no significant difference with the parental WT/pMTL84151 strain. These results suggest that the repression of toxin production by cysteine might be attributable to the accumulation of cysteine by-products, especially H_2_S. We also mapped a δ^54^-dependent promoter upstream from the *cdsB* gene, and the transcription of *cdsB* requires both δ^54^ and the CdsR protein in the presence of cysteine. Therefore, we propose that in response to excess cysteine, δ^54^ and its activator CdsR recognize their conserved sequence independently in the *cdsB* promoter and control the transcription of *cdsB*, and that strong expression of the CdsB protein accelerates the degradation of cysteine in the cells and produces a large amount of H_2_S, which can lead to a low level of toxin production. We proposed that the derepression of toxin production by cysteine in the *sigL* mutant might be attributable to an absence of transcription of the *cdsB* gene and the consequent lack of CdsB protein *in vivo*.

An analysis of inactivation in the *cdsB* gene also showed that CdsB enhances the tolerance of *C. difficile* for cysteine. Because free cysteine is cytotoxic, its intracellular concentration is stringently controlled (Sørensen and Pedersen, 1991; Hennicke et al., 2013). Cells remove redundant cysteine in two ways, degradation and export. In *Pantoea ananatis*, the *ccdA*-encoded desulfhydrase and the *cefA*-encoded cysteine efflux pump are directly involved in conferring resistance to excess levels of cysteine (Takumi and Nonaka, 2016). The deletion of the *cdsH* gene in *S. enterica* caused increased sensitivity to cysteine toxicity (Oguri et al., 2012). The ATP-binding cassette transporter CydDC in *E. coli* displays efflux activity, mediates cysteine transport from the cytoplasm to the periplasm, and ultimately exports excess cysteine (Pittman et al., 2002). We consider that CdsB in *C. difficile* might also function as a safety valve that maintains cysteine homeostasis when the intracellular cysteine concentration fluctuates. In the presence of excess cysteine, the strong expression of CdsB could help reduce toxicity and reverses the growth inhibition caused by cysteine.

Bacteria usually have a primary sigma factor that allows RNA polymerase to recognize the majority of promoters. However, in response to specific environmental stresses, they can also express multiple sigma factors that confer distinct promoter selectivity and form multiple holoenzymes (Gruber and Gross, 2003). The *sigL* gene encodes the sigma factor δ^54^ which activates genes involved in the utilization of nitrogen and carbon for energy and a range of other cellular processes in bacteria (Merrick, 1993; Buck et al., 2000; Francke et al., 2011). With further experiments and analyses, we showed that in response to cysteine, the expression of *cdsB* was activated by both the SigL and CdsR proteins. The CdsR protein shares strong similarity with δ^54^-dependent activators that contain the conserved “GAFTGA” amino acid sequence and the three essential functional domains, which suggests that the CdsR protein is the EBP^54^ activator of *cdsB*. To begin transcription, the RNA polymerase must interact with the EBPs bound to the UAS, which is located about 100–150 bp from the transcription start site (Francke et al., 2011). Based on the different activities of our fusion constructs, we suggest that the inverted repeat sequence that occurs in the region 153–223 bp upstream from the TSS is the UAS required for EBP^54^ binding.

In other microorganisms, such as *Salmonella* and *Yersinia*, a *cdsA*/*cuyP* gene encoding a cysteine transporter is reported to occur next to the *cdsB*/*cuyA* gene, which encodes a protein involved in the uptake of cysteine (Méndez et al., 2011; Loddeke et al., 2017). Before cysteine is degraded in cells, a cysteine transporter is required to import it into the cell and present it to a cysteine desulfidase. However, no ortholog of CuyP seems to be present in *C. difficile*, so other cysteine transporters might be involved in the uptake of cysteine. We searched the genome of *C. difficile* and found three sulfur-related transporter operons: CD2172–2177, CD2989–2991, and CD1482–1484. Based on our RNA-seq data for *C. difficile* R20291, the gene cluster CD2172–2177, which is similar to the *yxeI* operon of *Bacillus subtilis*, was significantly repressed in response to cysteine whereas the expression of the CD2989–2991 operon, which shares similarities with the alkanesulfonate ABC transporter *ssuABC* was upregulated in response to cysteine (data unpublished). Therefore, we propose that these two operons may be closely associated with the transport of cysteine, and that warrant further study.

In conclusion, we identified CdsB as an inducible cysteine desulfidase in *C. difficile* and demonstrated that the cysteine-dependent repression of toxin production in *C. difficile* was abolished by the inactivation of *cdsB*. We also compared the effects of cysteine by-products (H_2_S, pyruvate and NH_4_Cl) on toxin production and indicate that Na_2_S could significantly repress the toxin synthesis in *C. difficile*. The expression of *cdsB* is controlled by δ^54^, and the EBP^54^ activator CdsR is also required for the transcription of *cdsB*. Future studies should clarify the molecular mechanism underlying the effect of H_2_S on toxin synthesis. We believe that our results provide a new framework for the future treatment and control of *C. difficile* infection.

## Author contributions

HG designed, performed the experimentation, data analysis and wrote the manuscript. YY, MW, and SC contributed to experimental work. HW, SL, and YM participated in designed the experimentation, data interpretation and statistical analysis. JW designed, analyzed the data and revised the manuscript. All authors read and approved the final manuscript.

### Conflict of interest statement

The authors declare that the research was conducted in the absence of any commercial or financial relationships that could be construed as a potential conflict of interest.

## Supplementary material

Figure S1 Southern blotting and PCR amplification analysis of mutant strains. **(A)** Southern blotting analysis. Lane 1, digestion products of the recombinant vector pMTL007-*cdsB*, used as the positive control; lane 2, digestion products of the *C. difficile* 630Δ*erm* genome; lane 3, digestion products of the *C. difficile* strain 630Δ*erm cdsB::ermB* genome; lane 4, digestion products of the *C. difficile* strain 630Δ*erm sigL::ermB* genome; lane M, DNA molecular-weight marker (23,130, 9,416, 6,557, 4,361, 2,322, 2,027 bp). **(B)** PCR amplification analysis. Lane 1, PCR products of the *C. difficile* strain 630Δ*erm cdsR::ermB* genome with primers *cdsR*-screen-F and *cdsR*-screen-R; lane 2, PCR products of the *C. difficile* strain 630Δ*erm cdsR::ermB* genome with primers *cdsR*-screen-F and EBS universal; lane M, DNA molecular-weight marker (10,000, 7,000, 4,000, 2,000, 1,000, 500, 250 bp).

Figure S2 Toxin neutralization assay. Supernatants obtained from *C. difficile* strain 630Δ*erm* cultured in TY or TYC broth, diluted 5000-fold, preincubated with anti-TcdA and anti-TcdB serum and added to monolayers of Vero cells in 96-well plates. Cytotoxicity was recorded after 24 h.

Table S1 Primers used in the study.
