# Supplementary figures and images for: Novel Cysteine Desulfidase CdsB Involved in Releasing Cysteine Repression of Toxin Synthesis in Clostridium difficile
Source: Front Cell Infect Microbiol. 2018 Jan 9;7:531. doi: 10.3389/fcimb.2017.00531 (PMC5767170; doi:10.3389/fcimb.2017.00531)

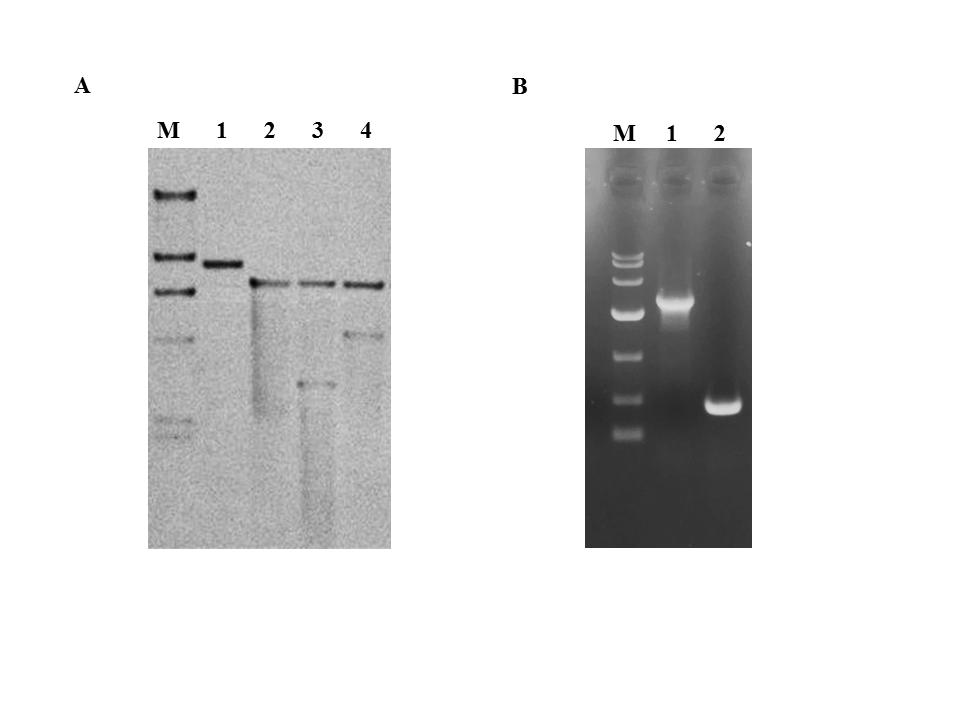

Supplement: Figure S1 — Southern blotting and PCR amplification analysis of mutant strains. (A) Southern blotting analysis. Lane 1, digestion products of the recombinant vector pMTL007-cdsB, used as the positive control; lane 2, digestion products of the C. difficile 630Δerm genome; lane 3, digestion products of the C. difficile strain 630Δerm cdsB::ermB genome; lane 4, digestion products of the C. difficile strain 630Δerm sigL::ermB genome; lane M, DNA molecular-weight marker (23,130, 9,416, 6,557, 4,361, 2,322, 2,027 bp). (B) PCR amplification analysis. Lane 1, PCR products of the C. difficile strain 630Δerm cdsR::ermB genome with primers cdsR-screen-F and cdsR-screen-R; lane 2, PCR products of the C. difficile strain 630Δerm cdsR::ermB genome with primers cdsR-screen-F and EBS universal; lane M, DNA molecular-weight marker (10,000, 7,000, 4,000, 2,000, 1,000, 500, 250 bp). [file Image1.TIF]

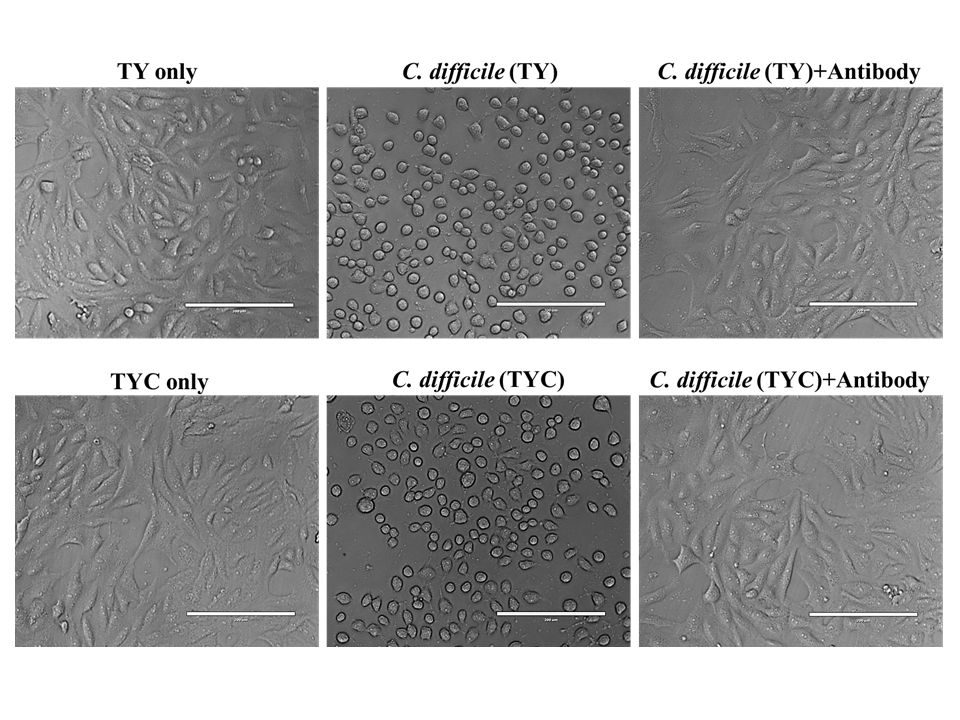

Supplement: Figure S2 — Toxin neutralization assay. Supernatants obtained from C. difficile strain 630Δerm cultured in TY or TYC broth, diluted 5000-fold, preincubated with anti-TcdA and anti-TcdB serum and added to monolayers of Vero cells in 96-well plates. Cytotoxicity was recorded after 24 h. [file Image2.TIF]
